# Sepsis: impact of timely and appropriate empirical antibiotic therapy on mortality

**DOI:** 10.1186/cc13548

**Published:** 2014-03-17

**Authors:** L Serpa-Pinto, T Cardoso

**Affiliations:** 1 Oporto Hospital Center, Oporto, Portugal

## Introduction

The administration of timely and appropriate antibiotic therapy is a well-known prognostic factor among severe sepsis patients [1-4]. The purpose of this study is to describe the magnitude of the impact of early and appropriate empirical antibiotic therapy on hospital mortality.

## Methods

A retrospective cohort study including all adult patients with sepsis admitted to the emergency room of a tertiary care, university hospital between 1 July 2011 and 30 June 2012.

## Results

A total of 1,219 patients were admitted to the emergency room during the study period, of which 162 (13%) had severe sepsis. Forty (25%) patients had withheld therapeutic decisions and were excluded from the current analysis; 20 additional patients transferred from other acute healthcare facilities were excluded due to missing or inaccurate data, leaving 102 patients to be included with a hospital mortality rate of 45%. The median time to antibiotics administration was 36 minutes (IQR 0 to 174), 59 (58%) patients had antibiotic administered within the first hour after sepsis recognition; 60 (59%) had positive microbiology, 74% with appropriate empiric antibiotic therapy. An association was found for hospital mortality with: heart failure (Or = 4.297; *P *= 0.037), decreased functional status (Karnofsky performance status <70%) (OR = 2.368; *P *= 0.034) and SOFA score (OR per point = 1.415; *P *< 0.001). Two multivariate models with hospital mortality as the dependent variable were built using alternative severity scores: one with SAPS II that was retained in the final model (adjusted OR = 1.068, 95% CI = 1.020 to 1.119) along with heart failure (adjusted OR = 5.859, 95% CI = 0.996 to 34.474); and another with SOFA score that was also retained in the final model (adjusted OR = 1.659, 95% CI = 1.227 to 2.242) along with heart failure (adjusted OR = 12.636, 95% CI = 1.423 to 112.229).

## Conclusion

Contrarily to what has been described previously, early and appropriate empirical antibiotic therapy was not associated with better prognosis. The most probable explanation is the higher compliance found with the current recommendations, reinforcing the need for period audits and feedback to the team.
